# The Brooklyn Treatment of Diphtheria

**Published:** 1879-10

**Authors:** Paul H. Kretzschmar


### SELECTIONS.

### THE BROOKLYN TREATMENT OF DIPHTHERIA.

BY PAUL H. KRETZSCHMAR, M. D.,

"Alcohol is as antagonistic to diphtheria as belladonna to opium, or quinine to malaria."—E. N. CHAPMAN, M. D.

In the *Hospital Gazette*, of June 14th, is a very interesting communication, in which the writer aims "to speak briefly of the merits of alcohol in the treatment of diphtheria, and to report his experience in thirty cases." The alcoholic treatment of diphtheria has been named the Brooklyn treatment, inasmuch as Dr. E. N. Chapman, of that city, as early as 1863, published his opinion on this subject. He has defended his assertions since that time on several occasions, and has lately published a valuable little work on the subject.

Dr. Chapman in his first article, published in the *Boston Medical and Surgical Journal*, reported nineteen recoveries in twenty successive cases, and during a period of three years, from early in 1874 to October, 1877, he had treated eighty-five cases of diphtheria by large doses of alcohol, with only one death. In all he reports one hundred and twenty-five cases, *with only two deaths*. If compared with the usual mortality of the disease, the statistics given are so remarkable as almost to stagger belief, more especially as the doctor has not even lost a case of croup during the period of his observation. Having tried the alcoholic treatment in thirty cases, I can confirm what Dr. Chapman says about its great efficiency and value. Of the thirty cases treated by this method, many of them were of the most severe variety, and a number of them complicated by scarlet fever, and I only lost four. If added to the statistics reported by Dr. Chapman, the number will aggregate one hundred and fifty-five cases; and of these one hundred and forty-nine recovered, and six died, a mortality of about four per cent. Whatever the true pathology of diphtheria may be; whether the local affection is only a symptom of a specific condition of blood-poisoning, or whether the disease should be regarded as a contagious infectious one which commences as a local difficulty, and which may either continue and remain such, or may lead secondarily to a constitutional affection, a large clinical experience shows that there is no form of treatment more effectual than the administration of large doses of alcohol, which does not act as a stimulant, and is not used to relieve or prevent great prostration, but simply for *its specific action as an antidote to the diphtheritic poison.* If administered early and in sufficient doses, alcohol acts as a preventive, its prophylactic properties being easily demonstrated. And even to cut short and prevent any of the sequelae of diphtheria, there is no remedy as reliable as alcohol. In most of Dr. Chapman's cases, quinine had been administered in addition to alcohol; in twelve of mine, all of which recovered, alcohol alone was used. The effect seemed to be almost as decided and quick without as with quinine. Lately I have always used quinine by inunction, and given the alcohol separately. I have in cases of diphtheria produced cinchonism by the frequent use of an ointment made of

Quiniae Sulp., ʒss.

Chloroformi, ʒss.

Bals. Peru, ʒss.

Adipis, ʒvi.

M.

For the administration of the alcohol, the following formula has been generally adopted:

Spts. vini gallici
vel. Spts. frumenti, ℥iiss.
Glycerini opt.
Syr. simplicis āā ℥ss.
Aquae menth. pip., q s ad ℥iv.
M.

To children under two years, ʒi every hour; from two to three years, ʒiss; from three to five years, ʒii; from five to eight years, ʒiiss to ℥ss every hour.

In a very few severe cases champagne has been used. Great importance is attached to hygiene; cleanliness and fresh air is obtained first of all.

Whether any good effect can be obtained from the topical treatment will not be discussed. I have always used a light astringent application, either in the form of a gargle or spray, or as a powder blown on the diseased parts; chlorate of potash, alum and tannic acid, with carbolic acid, have been used. Of late Wyeth's tablets of chlorate of potash have been found a convenient substitute.

The experience gained in thirty cases strongly impresses the facts that at least in diphtheria there is no kind of food so well borne by the stomach as lime water and milk. Dialysed iron has been used during the convalescence, and in combination with some form of alcohol, prevented the conditions of anaemia and loss of nerve power which frequently follow diphtheria. A few cases may be cited:

Walter B., aged 7, a rather delicate boy of healthy parents, attended school as usual during forenoon of April 4th, 1878. He had a very severe chill at 11:00 A. M., and went to bed after coming home at noon; I saw the boy at 4:00 P. M.; temperature 105½; pulse 134; skin dry; fauces coated with diphtheritic membrane; great prostration. I ordered brandy in teaspoonful doses every hour; cold application to head, and a carbolated chlorate of potash gargle. At 11:00 P. M. patient delirious; temperature 106¼; pulse 140; cold compresses continued, and brandy continued to ʒiss. every hour.

April 5th. Patient had a restless night; at 10:00 A. M. he feels quite comfortable, with a temperature of 101½; pulse 108; diphtheritic exudation extending. At 6:00 P. M. difficulty of breathing marked, the child struggling as in membranous croup; the administration of two doses of 3 grains each of turpeth mineral had the desired effect and caused free emesis; treatment continued.

10:00 P. M. Patient feels easier; dyspnoea diminished; temperature 102½; treatment continued.

April 6th, 10:00 A. M. Patient slept some; temperature 101½; a copious purulent secretion, followed by the disintegration of the membrane, took place; pulse very feeble. Under the "alcoholic treatment" the boy improved slowly, and was discharged free from any sequelae on April 22, 1878.

Louise H., aged 8, was taken sick with diphtheria in January, 1878. The origin of this case could be traced to sewer gas. Two other children, and the parents, escaped the disease by the exclusive use of alcohol and cinchonia, administered in the following way:

℞ Cinchon sulp., ʒi.
Acid sulp. arom., q. s.
Spts. Vini Gallici, ℥vii.
Glycerini opt., ℥i.
M.

From a small teaspoonful to a tablespoonful given every two hours, according to age.

In this case, the false membrane covered almost the entire pharynx. It soon extended both into the nostrils and downwards; the larynx became implicated to such an extent that tracheotomy was seriously considered. Two teaspoonful doses of brandy, given every hour, day and night, did not seem to influence the circulation at all, producing none of the common effects of alcohol, but carried the little patient safely through a very severe attack. The fetid discharge from the nose continued for about twelve days. No sequelae.

The writer reports five cases of scarlet fever, complicated with diphtheria in the same house, all of which were treated with alcohol and quinine with a successful result in each case. Inasmuch as the same principle is involved in the pathology and therapeutics of these cases, we leave our readers to judge of the value of the treatment from what has been written above.

